# Cigarette Smoke Induction of Interleukin-27/WSX-1 Regulates the Differentiation of Th1 and Th17 Cells in a Smoking Mouse Model of Emphysema

**DOI:** 10.3389/fimmu.2016.00553

**Published:** 2016-12-05

**Authors:** Shi-Lin Qiu, Min-Chao Duan, Yi Liang, Hai-Juan Tang, Guang-Nan Liu, Liang-Ming Zhang, Chao-Mian Yang

**Affiliations:** ^1^Department of Respiratory Medicine, The First Affiliated Hospital of Guangxi Medical University, Nanning, China; ^2^Department of Respiratory Medicine, The Fifth Affiliated Hospital of Guangxi Medical University and The First People’s Hospital of Nanning, Nanning, China

**Keywords:** COPD, cigarette smoke exposure, IL-27/WSX-1, Th1 cells, Th17 cells

## Abstract

IFN-γ-producing CD4^+^ T (Th1) cells and IL-17-producing CD4^+^ T (Th17) cells play a critical role in the pathogenesis of chronic obstructive pulmonary disease (COPD). However, the immune regulation between Th1 and Th17 cells remains unclear. Previous studies have demonstrated that interleukin-27 (IL-27)/WSX-1 exerted pro- or anti-inflammatory effects in many acute inflammatory diseases by modulating T cell-mediated immune response, but little was known about its role in chronic inflammatory disease, especially in smoking-related lung diseases. Considering IL-27 is an important regulator in T lymphocytes immune responses and was found markedly increased in patients with COPD, we hypothesized that IL-27/WSX-1 may exert immuno-regulatory effects on the differentiation of Th1 and Th17 cells in smoking-related COPD. In this study, we aimed to evaluate the expression of IL-27 in patients with COPD and explore the role of IL-27/WSX-1 on Th1 and Th17 cells differentiation in a smoking mouse model of emphysema. We found that elevated expression of IL-27 was associated with increased proportion of Th1 cells and Th17 cells in patients with COPD and demonstrated parallel findings in cigarette smoke-exposed mice. In addition, cigarette smoke exposure upregulated the expression of IL-27R (WSX-1) by naive CD4^+^ T cells in mice. *In vitro*, IL-27 significantly augmented the secretion of IFN-γ by naive CD4^+^ T cells *via* a T-bet, p-STAT1, and p-STAT3-dependent manner, but inhibited the production of IL-17 by a ROR-γt and p-STAT1-dependent way. Furthermore, anti-IL27 treatment dramatically decreased the expression of IFN-γ-producing CD4^+^ T cells in cigarette smoke-exposed mice. These findings proposed that IL-27 has functions for promoting the expression of Th1 cells but inhibiting the expression of Th17 cells *in vitro* and IL-27 neutralization-attenuated Th1-mediated inflammation *in vivo*, suggesting targeting IL-27/WSX-1 may provide a new therapeutic approach for smoking-related COPD.

## Introduction

Chronic obstructive pulmonary disease (COPD) is characterized by chronic airway inflammation and destruction of lung parenchyma. Tobacco smoking is recognized as the most important risk factor for COPD. Despite increased awareness and concerns of smoking hazards in general population, the global burden of smoking-related lung diseases (e.g., COPD, chronic bronchitis, and lung cancer) continues to increase in worldwide. Large studies have demonstrated that the mortality among smokers who began to smoke in early adult life and did not quit was two to three times the mortality among those who never smoked, leading to a reduction in life span by an average of about 10 years (1–5). Although COPD is expected to become the third leading cause of death worldwide by 2020 and imposes a heavy burden on health-care systems, the precise pathogenesis of smoking-related COPD has not yet been fully elucidated. It has been well established that COPD exhibited a predominant IFN-γ-producing T (Th1/Tc1) cell cytokine pattern (6–8). Interestingly, Th1 cells are generally in association with IL-17-producing CD4^+^ T helper (Th17) cells in the context of infectious or some autoimmune diseases, such as experimental autoimmune encephalitis (EAE), collagen-induced arthritis (CIA), and inflammatory bowel disease (IBD), which typically with Th1-mediated pathogenesis. In our recent studies and with others, data revealed that Th17 cells were also exaggerated in COPD (9–11). However, relatively little is known about the regulatory mechanism between Th1 and Th17 cells.

Interleukin-27 (IL-27) is a heterodimeric cytokine composed of EBI3 and p28 and produced mainly by activated antigen-presenting cells (APCs), such as macrophages and dendritic cells (12). IL-27 binds to a receptor complex called IL-27R, which is composed of WSX-1 and gp130 (13). Despite the expression of gp130 is widely on various immune and non-immune cells, WSX-1 is more confined to immune cells such as T cell, B cell, and NK cell. IL-27 not only exerts pro-inflammatory activity by enhancing Th1 response but also has potential anti-inflammatory function able to regulate excessive Th1 response *via* increasing the production of IL-10 by Th1 cells and inhibition of IL-2 secretion (14–19). Aside from the effects on Th1 cells, IL-27 has recently reported that played a regulatory role in limiting excessive inflammation during infection with *Toxoplasma gondii* by modulating the development of Th17 cells (20, 21). Moreover, recent studies proposed a pivotal role of IL-27 in enhancing regulatory T cells (Tregs) function to control T cell-induced colitis, a model for IBD in humans (18, 22). Although IL-27/WSX-1 has been found to participate in many acute inflammatory diseases such as bacterial and parasitic infections, but very little information was available concerning its involvement in chronic inflammatory disease, especially in smoking-related lung diseases. More recently, Cao et al. reported that IL-27 was elevated in patients with COPD and IL-27 level in Sputum was correlated negatively with FEV1 (% pred), suggesting a critical role of IL-27 in the pathogenesis of COPD (23). Given IL-27 is an important regulator in T lymphocytes immune responses and was found markedly increased in patients with COPD, it is reasonable to predict IL-27/WSX-1 may involve in the immune regulation of Th1 and Th17 cells in COPD. In the present study, we sought to evaluate the expression of IL-27 and Th1/Th17 cells in patients with COPD and then explore the role of IL-27/WSX-1 on Th1 and Th17 cells differentiation in a smoking mouse model of emphysema.

## Materials and Methods

### Subjects

Thirty-eight patients with stable COPD, 20 healthy smokers, and 20 healthy non-smoking control subjects were enrolled in this study. COPD patients were diagnosed according to the criteria as defined by the Global Initiative for Chronic Obstructive Lung Disease (GOLD), were with a smoking history of at least 10 pack-years. Patients with COPD who had an acute exacerbation for 4 weeks before enrollment were excluded. Patients who received systemic corticosteroids, leukotriene antagonists, or antihistamines within the month prior to the study were also excluded. This study was approved by the Medical Ethical Committee of the First Affiliated Hospital of Guangxi Medical University (Nanning, China) and the Medical Ethical Committee of the Eighth People’s Hospital of Nanning (Nanning, China), and written informed consent was obtained from all participants. Clinical characteristics of patients with COPD and control subjects are presented in Table 1.

**Table 1:** **Patient characteristics**.

Characteristics	Non-smokers (*n* = 20)	Healthy smokers (*n* = 20)	COPD (*n* = 38)
Age, years	63 ± 9	60 ± 10	64 ± 9
Sex, M (F)	10 (10)	18 (2)	29 (9)
Smoking history (pack-years)	0 ± 0	32 ± 10	36 ± 21
Smoking status, current (former)	0 (0)	20 (0)	16 (22)
FEV 1, l	2.7 ± 0.3	2.6 ± 0.3	1.2 ± 0.2^a,b^
FVC, l	3.3 ± 0.4	3.4 ± 0.3	2.7 ± 0.5^c,d^
FEV1 (% pred)	97.0 ± 5.2	96.9 ± 4.3	41.7 ± 5.6^e,f^
FEV 1/FVC, %	80.6 ± 4.1	76.9 ± 3.4	46.9 ± 6.1^g,h^
Taking inhaled steroids	0	0	0

*Data are expressed as means ± SD; statistical analysis was performed with one-way ANOVA*.

*M, male; F, female; FEV1 (% pred), forced expiratory volume in 1 s (% of predicted)*.

*^a,c,e,g^p < 0.05 when compared with non-smokers*.

*^b,d,f,h^p < 0.05 when compared with healthy smokers*.

### Human Blood Sample Collection and Processing

Ten milliliters of venous blood were collected from each subject, and serum was isolated by centrifugation for 15 min at 800 × *g* and stored at −80°C immediately until analysis. To obtain peripheral blood mononuclear cells (PBMCs), blood samples were anti-coagulated with heparin and separated by Ficoll-Hypaque gradient centrifugation. Fresh isolated PBMCs were kept on ice and used for flow cytometric analysis within 1 h.

### Animals and Cigarette Smoke Exposure

Male BALB/c mice (8 weeks of age, 20–25 g body weight) were purchased from the Guangxi Medical University Laboratory Animal Center (Nanning, China). All mice were housed in sterilized cages and maintained on a 12:12-h light–dark environment and received sterilized diet and water *ad libitum*. All animal studies were approved by the Laboratory Animal Ethics Committee of Guangxi Medical University.

Mice were exposed to cigarette smoke, as described previously (11). Briefly, mice were exposed to five cigarettes (Nanning zhenlong unfiltered cigarettes: 12 mg of tar and 0.9 mg of nicotine) four times a day with 30-min smoke-free intervals in a closed 0.75-m^3^ room, 5 days a week, for 24 weeks. Mice tolerated cigarette smoke exposure without evidence of toxicity (carboxyhemoglobin levels ~10% and no weight loss). The control groups were exposed to air for 24 weeks. The mice were sacrificed 24 h after the last air or smoke exposure.

### Mouse Sample Collection and Processing

Twenty-four hours after the last exposure, mice were anesthetized with pentobarbital. Blood samples were collected *via* retro-orbital bleeding. Approximately 300 μl of peripheral blood was collected for separate serum after centrifugation (800 × *g*, 15 min), and serum was frozen at −80°C immediately for determination of the concentration of cytokines. The remaining blood was collected in EDTA-treated tubes and was divided into two parts. Approximately 100 μl of peripheral blood was removed *via* erythrocytes with RBC lysis solution for 10 min at room temperature and then washed twice with cold PBS by centrifuged at 300 × *g* for 10 min. The remaining cells were collected for isolation of total RNA. PBMCs were isolated by Ficoll-Hypaque gradient centrifugation for flow cytometry.

In order to obtain spleen cells suspension, spleens were removed and cut into small pieces and were subsequently ground gently into single-cell and filtered through nylon mesh. The cell suspension was centrifuged at 300 × *g* for 10 min at 4°C. After that, the erythrocytes of cells suspension were removed, as described previously, and the cell pellets of spleen were washed twice with cold PBS.

Lung single-cell suspensions were isolated by modifying established protocols, using a combination of mechanical fragmentation, enzyme digestion, and centrifugation procedures, as described previously (8, 24). Briefly, lungs were flushed *via* the right ventricle with a needle and the pulmonary circulation was perfused with 10 ml of PBS to remove the intravascular pool of cells. The lungs were then thoroughly minced and followed by a 30- to 45-min digestion at 37°C with 1 mg/ml collagenase type IV (Sigma-Aldrich) in RPMI 1640 medium. To improve tissue disintegration, lungs were incubated in a shaker and pipetted every 15 min. Subsequently, the lung pieces were triturated through a metal screen into PBS with a plunger from a 5-ml syringe, and the resulting cell suspension was filtered through a 70-μm cell strainer, washed, and lung mononuclear cells were isolated by Ficoll-Hypaque gradient centrifugation. The cell suspension was then resuspended in PBS and kept on ice until labeling.

### Histology and Morphometry Assay

The lungs were fixed with 10% formalin, embedded in paraffin, cut sagittally into 5-μm sections, and stained with hematoxylin and eosin (H&E) for histological analysis. We examined the enlargement of alveolar spaces by quantifying the mean linear intercept (Lm), as described previously. For each animal, 10 fields at a magnification of 200× were captured randomly from the four different zones of the lung. Two investigators blinded to the exposure status independently measured Lm. Two investigators independently measured Lm in a blinded manner.

### Flow Cytometry

The expression markers on T cells from PBMC, spleen, and lung were determined by flow cytometry using the following antibodies: PerCP-CD4, PE-CD62L, APC-CD44, FITC-IL27R (WSX-1), PE-IL17, and APC-IFN-γ, which were purchased from BD Pharmingen (San Diego, CA, USA) or R&D systems (Minneapolis, MN, USA). Cell surface staining was performed according to the standard procedures. For intracellular detection of cytokines, cells were stimulated with phorbol-myristate-acetate (PMA, 25 ng/ml; Sigma-Aldrich) and ionomycin (1 ng/ml; Sigma-Aldrich) in the presence of GolgiPlug™ (BD Pharmingen) for 4 h at 37°C in 5% CO_2_. The cells were then washed and stained with fluorescent antibodies against CD4 and IL27R (WSX-1) at room temperature in the dark. After surface staining, cells were fixed/permeabilized in fixation/permeabilization solution (Cytofix/Cytoperm™; BD Pharmingen) according to the manufacturer’s protocol, and stained with anti-IFN-γ and -IL-17 mAbs for 30 min at 4°C. Cells were then washed with Perm/Wash Buffer (BD Pharmingen) and resuspended in PBS +2% FBS for flow cytometric analysis. Flow cytometry was performed on a BD FACS Canto II (BD Biosciences) and analyzed using FCS Express 4 software (*De Novo* Software, Los Angeles, CA, USA).

### Real-time Quantitative PCR

For quantifying the relative transcript abundance of IL-27 (EBI3 and p28), total RNA was extracted from peripheral blood, spleens, and lung samples with TRIzol (Invitrogen, Life Technologies) according to the manufacturer’s instructions. cDNA was prepared using oligo(dT) primers (PrimeScript™RT reagent Kit, TAKALA). Quantitative RT-PCR was performed by duplicate with SYBR Green I (SYBR^®^Premix Ex Taq™, TAKALA) using an Applied Biosystems 7500 (ThermoFisher SCIENTIFIC) according to the manufacturer’s instructions. DNA was amplified under the following conditions: denaturation at 95°C for 30 s, extension at 95°C for 5 s, 60°C for 34 s, and the samples were amplified for 40 cycles. The following primers were used: 5′-TGTGTCCGTCGTGGATCTGA-3′, 5′-TTGCTGTTGAAGTCGCAGGAG-3′ for GAPDH; 5′-TGCCAGGAGTGAACCTGGAC-3′ and 5′-CCGAAGTGTGGTAGCGAGGA-3′ for P28; 5′-AGAGCCACAGAGCATGTCCAA-3′ and 5′-TGCACTCTGGGCTGGCTTAG-3′ for EBI3. The band sizes of the fragments were 125 bp (P28), 111 bp (EBI3), and 149 bp (GAPDH). GAPDH was used as an internal control, and levels of each gene were normalized to GAPDH expression using the ΔΔ*C*_t_-method. The identity of the amplified products was examined using agarose gel electrophoresis s and melt curve analysis.

### Cytokine Measurement

The concentrations of IL-27 in serum and the lungs were measured by ELISA kits according to the manufacturer’s protocols (CUSABIO, Wuhan, China). All samples were assayed in duplicate.

### Cell Isolation

Spleen cells suspension was obtained, as described above. Naive CD4^+^ T cells from suspensions of spleen were isolated by Naive CD4^+^ T Cell Isolation Kit (Miltenyi Biotec, Auburn, CA, USA) according to the manufacturer. Briefly, non-naive CD4^+^ T cells including cytotoxic T cells, Tregs, activated T cells, B cells, NK cells, macrophages, granulocytes, endothelial cells, and erythroid cells were indirectly magnetically labeled by using a cocktail of biotin-conjugated antibodies and Anti-Biotin MicroBeads. Memory T cells were directly magnetically labeled with CD44 MicroBeads. Isolation of highly pure naive CD4^+^ T cells was achieved by depletion of magnetically labeled non-target cells. The purity of CD44^low^ CD62L^+^ naive CD4^+^ T cells was >90%, as measured by flow cytometry.

### Effects of IL-27 on the Differentiation of Th1 and Th17 Cells *In Vitro*

Purified naive CD4^+^ T cells were plated in 24-well plates (Costar) at a density of 1 × 10^6^ cells/ml in RPMI-1640 medium containing 10% FCS and were stimulated with plate-bound anti-CD3 (5 mg/ml; 145-2C11; eBioscience, San Diego, CA, USA) and soluble anti-CD28 (2 mg/ml; 37.51; eBioscience). Th1 polarizing conditions as follow: IL-12 (20 ng/ml; eBioscience) and anti-IL-4 (10 μg/ml; 11B11; eBioscience). For Th17 differentiation, cultures were supplemented with recombinant mouse IL-1β (10 ng/ml; Peprotech), IL-6 (20 ng/ml; eBioscience), IL-23 (20 ng/ml; eBioscience), TGF-β (2 ng/ml; eBioscience), anti-IL-4 mAb (10 μg/ml; 11B11; eBioscience), and anti-IFN-γ mAb (10 μg/ml; XMG1.2; eBioscience). Mouse recombinant IL-27 (eBioscience) was added to some samples at different concentrations (1–100 ng/ml). Cultures were also supplemented with IL-2 (10 ng/ml; eBioscience) on days 2 and 4, refresh cells cultured with new medium and reagents on days 3. After cultured for 6 days, some cells were harvested and stimulated with PMA (25 ng/ml; Sigma-Aldrich) and ionomycin (1 ng/ml; Sigma-Aldrich) in the presence of GolgiPlug™ (BD Pharmingen) for 4 h. The cells were subsequently stained with anti-IL-17 and anti-IFN-γ mAbs. To intracellular stain with anti-T-bet (eBio4B10; eBioscience) and anti-ROR-γt (B2D; eBioscience), some cells were permeabilized with Foxp3/Transcription Factor Staining Buffer Set (eBioscience) strictly according to the manufacturer’s instructions.

For assessing the effects of IL-27 on the cells after Th1 and Th17 polarization, cultures were supplemented with IL-27 at different concentrations (1–100 ng/ml) on days 6 and cultured for 2 more days, the cells were harvested after stimulated with PMA and ionomycin and stained with anti-IL-17, anti-IFN-γ, anti-T-bet, and anti-ROR-γt mAbs, as described above.

### Signal Transductions of IL-27 on Th1 and Th17 Differentiation

To examine the transcription factors involved in IL-27 regulating the differentiation of Th1 and Th17 cells, naive CD4^+^ T cells were stimulated at Th1 or Th17 polarization conditions, as described above. Three days later, the medium was supplemented with IL-27, and the cells were stimulated for 30 min and stained with PE-pSTAT1 (Tyr701), Alexa Fluor647 -pSTAT3 mAbs (Tyr705, all from Cell Signaling Technology, Inc., Danvers, MA, USA). Briefly, cells were fixed with 4% paraformaldehyde for 10 min at 37°C, permeabilized by incubate 30 min on ice with ice-cold 100% methanol. Cells were then incubated with p-STAT1 and p-STAT3 mAbs for 1 h at room temperature. Flow cytometry was performed, as mentioned above.

### Effects of IL-27 Neutralization in Cigarette Smoke-Exposed Mice

Mice were exposed to cigarette smoke for 24 weeks, as described above. The effect of IL-27 neutralization on mouse was examined by tail vein injection with anti-mouse IL-27 p28 functional grade purified mAbs (MM27-7B1; eBioscience) twice a week (10 μg in 200 μl PBS per time) in the course of cigarette smoke exposure. Morphometry assay and cells collection were managed with procedure, as described above.

### Statistics

Data were expressed as mean ± SD. Differences between groups were compared unpaired Student’s *t*-test or one-way ANOVA. Correlations between variables were determined by Spearman’s rank correlation test. Analysis was completed using SPSS version 16.0 Statistical Software (Chicago, IL, USA), and *p* < 0.05 was considered to indicate statistical significance.

## Results

### Elevated IL-27 Was Associated with Increased Expression of Th1 and Th17 Cells in Patients with COPD

**Figure 1:**
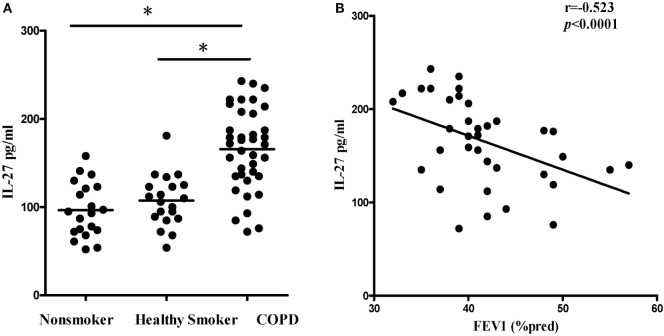
**Elevated expression serum levels of IL-27 in patients with COPD**. **(A)** IL-27 protein concentrations in serum of non-smokers, healthy smokers, and COPD patients. Data are expressed as means ± SD. *Horizontal bars* indicate means. The comparisons were determined by one-way ANOVA. *p < 0.05 compared with one another among three groups. **(B)** Correlation between serum IL-27 and FEV1 (% pred) in COPD patients. Correlations between two parameters were determined by Spearman’s rank correlation test.

We first quantified the concentrations of serum IL-27 in patients with COPD using ELISA. Compared with non-smokers or healthy smokers, serum IL-27 was significantly elevated in patients with COPD (96.5 ± 36.8 and 107.4 ± 29.0 and 165.6 ± 46.0 pg/ml, respectively; *p* < 0.05). However, there was no significant difference between non-smokers and healthy smokers (Figure 1A). In addition, serum IL-27 concentrations were correlated negatively with FEV1 (% pred) in patients with COPD (Figure 1B).

**Figure 2:**
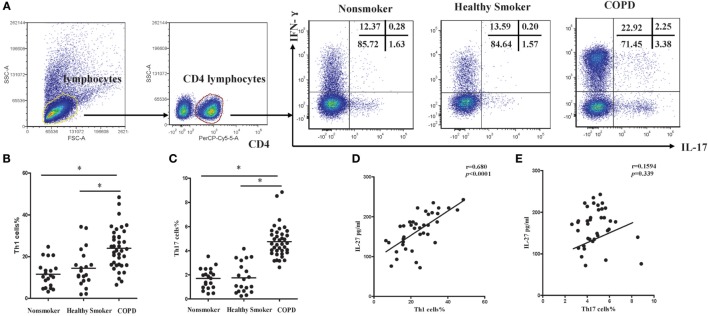
**Increased expression of Th1 and Th17 cells in peripheral blood of COPD patients**. **(A)** Lymphocytes were identified based on their characteristic properties shown in the FSC and SSC, and CD4^+^ T cells were gated from lymphocytes. The representative flow cytometric dot plots of Th1 and Th17 cells in peripheral blood of non-smokers, healthy smokers, and COPD patients. FSC, forward scatter cytometry; SSC, sides scatter cytometry. **(B)** Comparisons of percentages of Th1 cells **(C)** and Th17 cells in non-smokers, healthy smokers, and COPD patients. Data are expressed as means ± SD. *Horizontal bars* indicate means. The comparisons were determined by one-way ANOVA (*p < 0.05). **(D)** Correlation between serum IL-27 and Th1 **(E)** and Th17 cells in COPD patients. Correlations between two parameters were determined by Spearman’s rank correlation test.

Subsequently, we investigated the expression of Th1 and Th17 cells in patients with COPD. Flow cytometry was performed on PBMCs with gating on CD4^+^ T lymphocytes (Figure S1A in Supplementary Material). The representative flow cytometric dot plots of Th1 and Th17 cells in peripheral blood were showed in Figure 2A. When compared with non-smokers or healthy smokers, the percentages of Th1 cells showing a significant increase in patients with COPD (Figure 2B). In addition, we noted that the frequencies of Th17 cells were significantly higher in COPD patients than in non-smokers or healthy smokers (Figure 2C). We also found that IL-17^+^IFN-γ^+^CD4^+^ T cells were significantly higher in COPD patients. Furthermore, the levels of serum IL-27 in COPD patients were correlated positively with the percentages of Th1 cells (Figure 2D), suggesting functional involvement of IL-27 in regulating the differentiation of Th1 cells. However, there was no detectable correlation between serum IL-27 levels and the percentages of Th17 cells in peripheral blood of COPD patients (Figure 2E).

### Cigarette Smoke Exposure Induced Elevated IL-27 Expression in Mice

**Figure 3:**
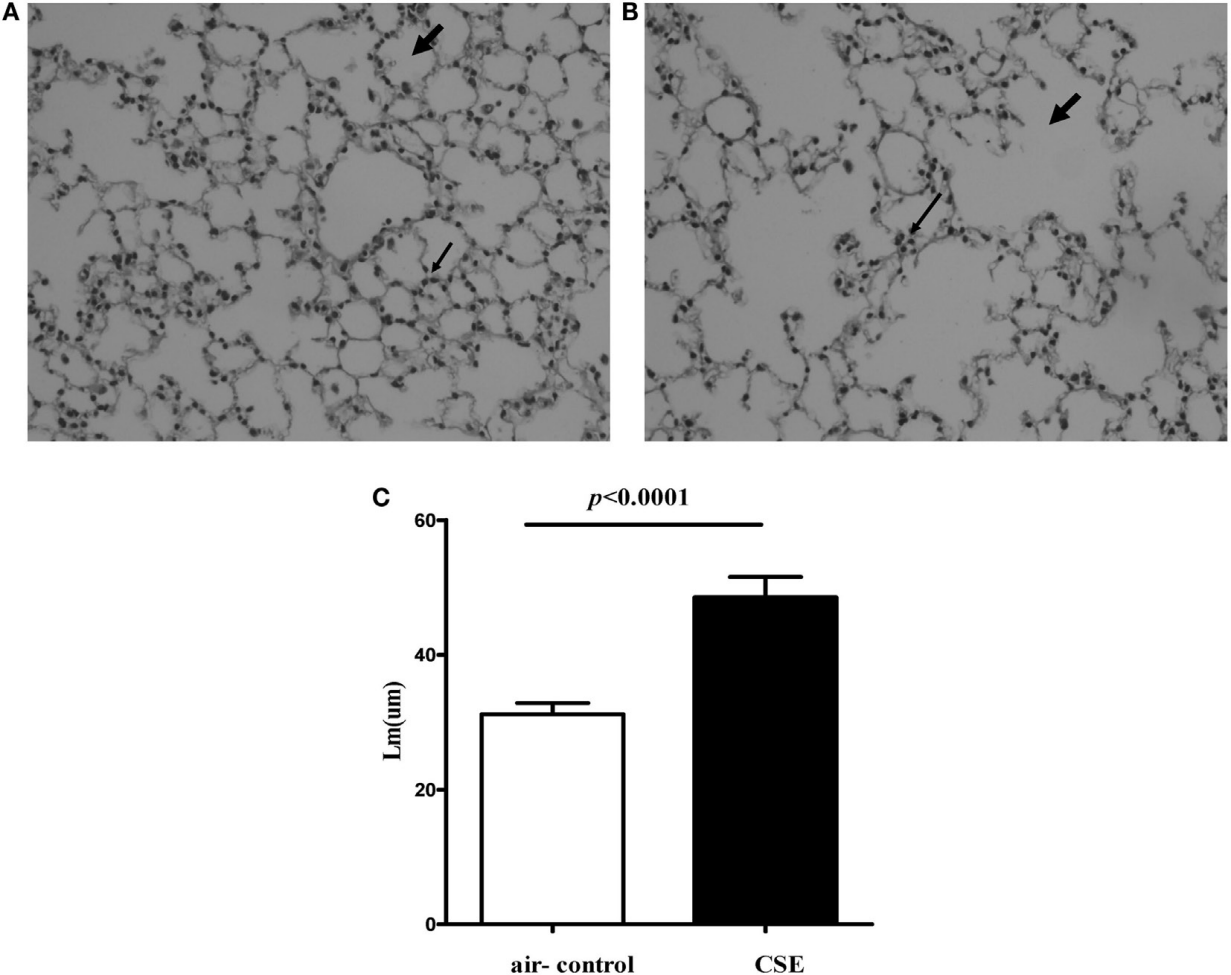
**Cigarette smoke exposure (CSE) induced alveolar destruction and airspace enlargement in mice**. Representative photomicrographs of hematoxylin and eosin-stained lung tissue of air-control and cigarette smoke-exposed mice at 24 weeks (magnification, ×200). **(A)** Air-control mice **(B)** cigarette smoke-exposed mice. The arrows in **(A)** indicate the normal alveolar and the lymphocytes in alveolar septa in air-control mice. The arrows in **(B)** represent the enlargement of the air spaces the destruction of the alveolar architecture accompanied by the infiltration of lymphocytes. **(C)** Comparisons of Lm values in air-control and cigarette smoke-exposed mice. *n* = 10 animals/group; data are expressed as means ± SD. The comparisons were determined by Student’s *t*-test.

Because cigarette smoking is critical to the pathogenesis of COPD, we assessed IL-27 in a smoking mouse model of emphysema. The mice developed an emphysematous phenotype after 24 weeks of cigarette smoke exposure, showing enlargement of the air spaces accompanied by the destruction of the alveolar architecture. The representative morphometric results of the lungs after cigarette smoke exposure were presented in Figures 3A,B. To quantify the presence and severity of emphysema, we determined the enlargement of alveolar spaces by measuring the mean linear intercept (Lm). Compared with air-control mice (31.2 ± 1.6 μm), significant air space enlargement was observed in cigarette smoke-exposed mice (48.5 ± 3.1 μm, respectively; *p* < 0.0001) (Figure 3C).

**Figure 4:**
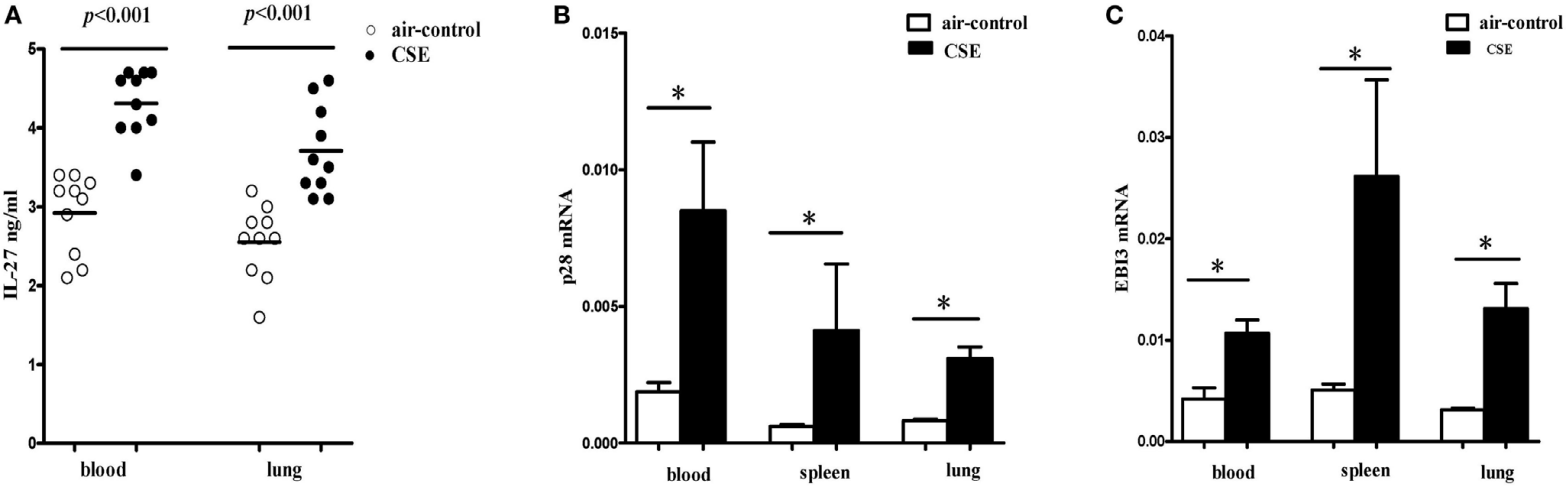
**Elevated expression levels of IL-27 in cigarette smoke-exposed mice**. **(A)** IL-27 protein concentrations in serum, lungs of air-control and cigarette smoke-exposed mice. **(B)** IL-27 p28 mRNA **(C)** and EBI3 mRNA expression in peripheral blood, spleens, and lungs of air-control and cigarette smoke-exposed mice. Data are expressed as means ± SD. The comparisons were determined by Student’s *t*-test (*p < 0.05).

We next detected the levels of IL-27 protein in cigarette smoke-exposed mice. The levels of IL-27 protein in blood and lung were measured with ELISA. Consistent with the results of COPD patients, we found that IL-27 protein was significantly upregulated in blood of mice after cigarette smoke exposure when compared with air-control mice. Similarly, the levels of IL-27 protein in lungs of cigarette smoke-exposed mice were also significantly higher than that of air-control (Figure 4A).

To further confirm our observations, the mRNA expression levels of p28 and EBI3 in blood and spleen and lung of cigarette smoke-exposed mice were quantified by quantitative RT-PCR. In tissues (blood, spleen, and lung) from cigarette smoke-exposed mice, we observed that the p28 mRNA expression was markedly higher than in air-control mice (Figure 4B). Similarly, there was a significant increase in EBI3 mRNA expression in tissues (blood, spleen, and lung) from cigarette smoke-exposed mice (Figure 4C).

### Cigarette Smoke Exposure Induced Upregulated IL-27R (WSX-1) Expression in Mice

**Figure 5:**
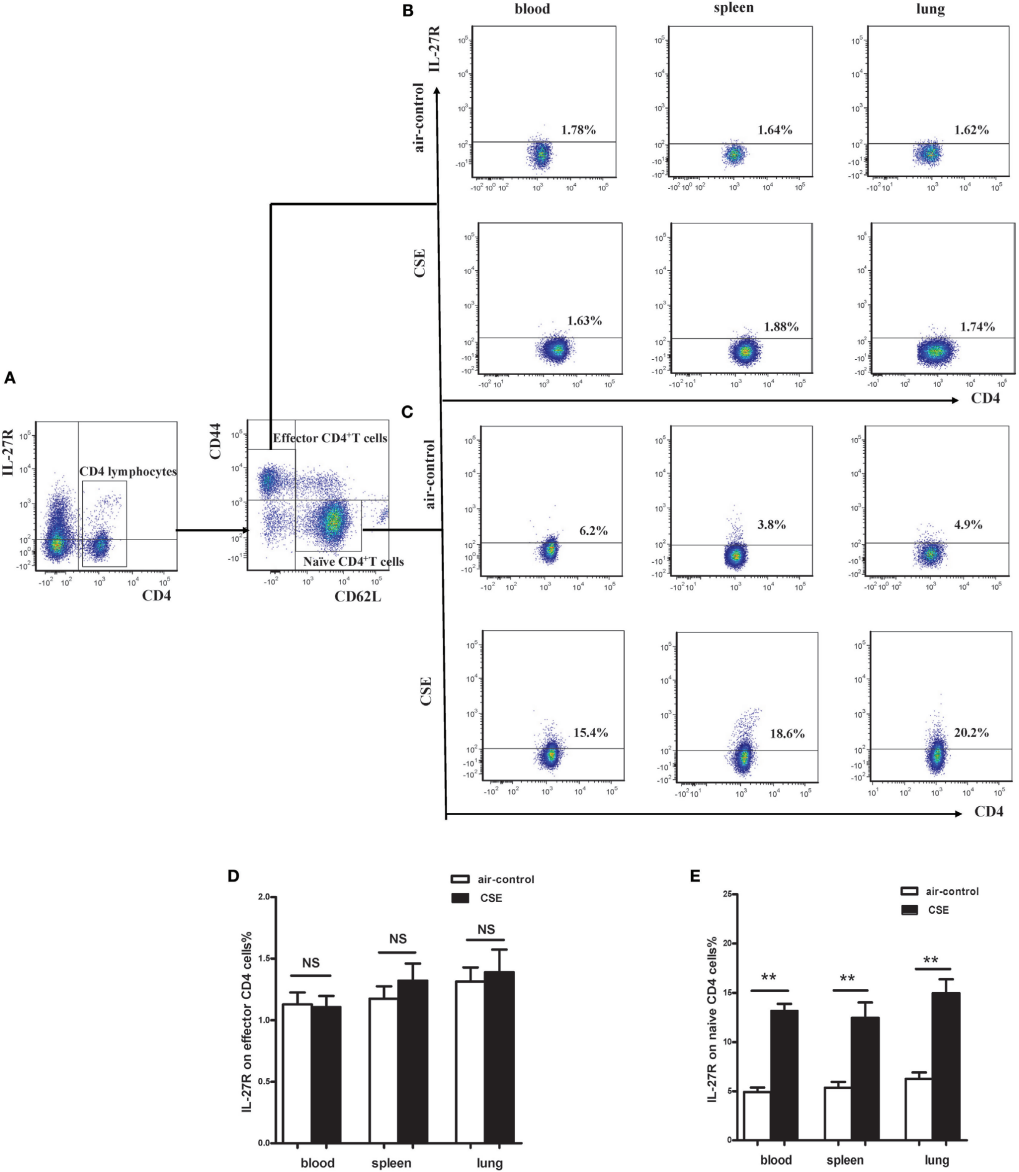
**Increased expression of IL-27R (WSX-1) on effector and naïve CD4^+^ T cells in cigarette smoke-exposed mice**. **(A)** The representative flow cytometric dot plots of IL-27R (WSX-1) on total CD4^+^ T cells. The expression of IL-27R (WSX-1) on **(B)** CD44^high^CD62L^−^effector CD4^+^ T cells **(C)** and CD44^low^CD62L^+^naïve CD4^+^ T cells in mice of air-control or cigarette smoke exposed. The comparison of IL-27R (WSX-1) expression on **(D)** effector CD4^+^ T cells **(E)** and naïve CD4^+^ T cells in peripheral blood, spleens, and lungs of air-control or cigarette smoke-exposed mice. NS, no difference. Data are expressed as means ± SD. The comparisons were determined by Student’s *t*-test (**p < 0.001).

Given the critical importance of IL-27R (WSX-1) in IL-27 signaling, we sought to investigate the expression of IL-27R (WSX-1) in cigarette smoke-exposed mice. As shown in Figure 5A, we found IL-27R (WSX-1) was expressed on total CD4^+^ T cells, which consistent with previous studies. Yet, the expression of IL-27R (WSX-1) on subgroup of CD4^+^ T cells has not been elucidated in detail. We then examined the phenotype of CD4^+^ IL-27R^+^ T cells and found that most of the CD4^+^IL-27R^+^ T cells were CD44^low^CD62L^+^naive CD4^+^ T cells, only a small proportion of CD44^high^ CD62L^−^ effector CD4^+^ T cells expressed IL-27R (data not shown). These results indicated that naive CD4^+^ T cells are the major target of IL-27 immune regulating function. We further examined the expression of IL-27R (WSX-1) on effector and naïve CD4^+^ T cells in cigarette smoke-exposed mice. Our result demonstrated that the expression of IL-27R (WSX-1) on effector CD4^+^ T cells did not show any differ between two group (Figures 5B,D). However, IL-27R (WSX-1) was notably upregulated on naïve CD4^+^ T cells from cigarette smoke-exposed mice, as compared with air-control mice (Figures 5C,E).

### The Phenotypic Characteristics of CD4^+^ T Cells and Proportions of Th1 and Th17 Cells in Mice of Cigarette Smoke Exposure

**Figure 6:**
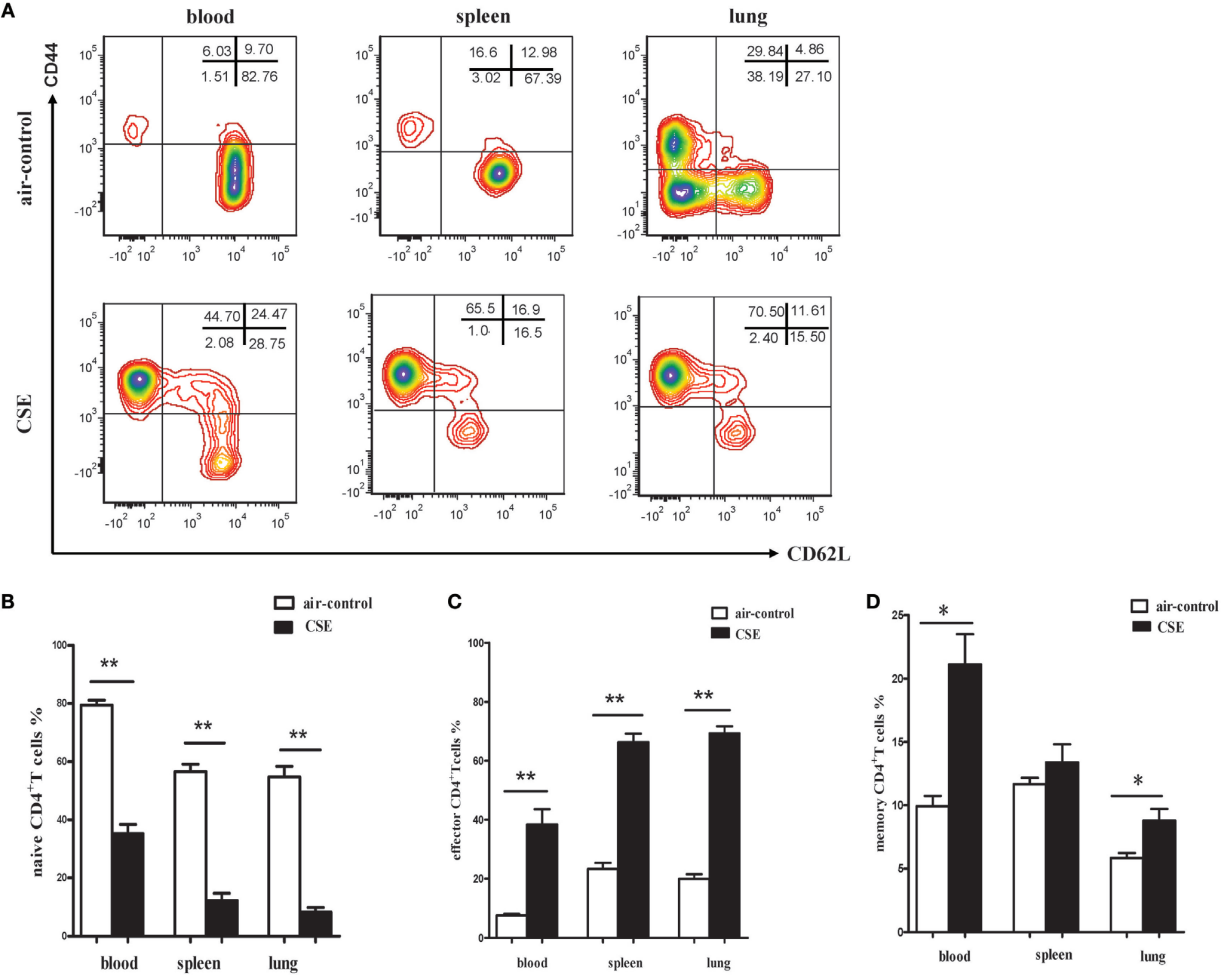
**Phenotypic characteristics of CD4^+^ T cells in cigarette smoke-exposed mice**. **(A)** The representative flow cytometric dot plots of naïve (CD44^low^CD62L^+^), memory (CD44^high^CD62L^+^), and effector (CD44^high^CD62L^−^) CD4^+^ T cells in peripheral blood, spleens, and lungs of air-control and cigarette smoke-exposed mice. **(B)** Comparisons of percentages of naïve CD4^+^ T cells **(C)** and effector CD4^+^ T cells **(D)** memory CD4^+^ T cells in air-control and cigarette smoke-exposed mice. Data are expressed as means ± SD. The comparisons were determined by Student’s *t*-test (*p < 0.01, **p < 0.001).

Since elevated IL-27 was observed in mice of cigarette smoke exposure and IL-27R (WSX-1) was mainly expressed on naive CD4^+^ T cells, we interested in the phenotypic characteristics of CD4^+^ T cells after cigarette smoke exposure. As shown in Figures 6A,B, a significant decrease of CD44^low^CD62L^+^ naive CD4^+^ T cells was observed in peripheral blood of cigarette smoke-exposed mice when compared with air-control mice. In contrast, the percentages of CD44^high^CD62L^−^ effector CD4^+^ T cells in peripheral blood were significantly increased after 24 weeks of cigarette smoke exposure (Figures 6A,C). Similar results were obtained in samples from spleens and lungs in cigarette smoke-exposed mice (Figures 6A–C). Moreover, the percentages of CD44^high^ CD62L^+^memory CD4^+^ T cells in peripheral blood and lungs of cigarette smoke-exposed mice were higher than that in air-control mice (Figure 6D).

**Figure 7:**
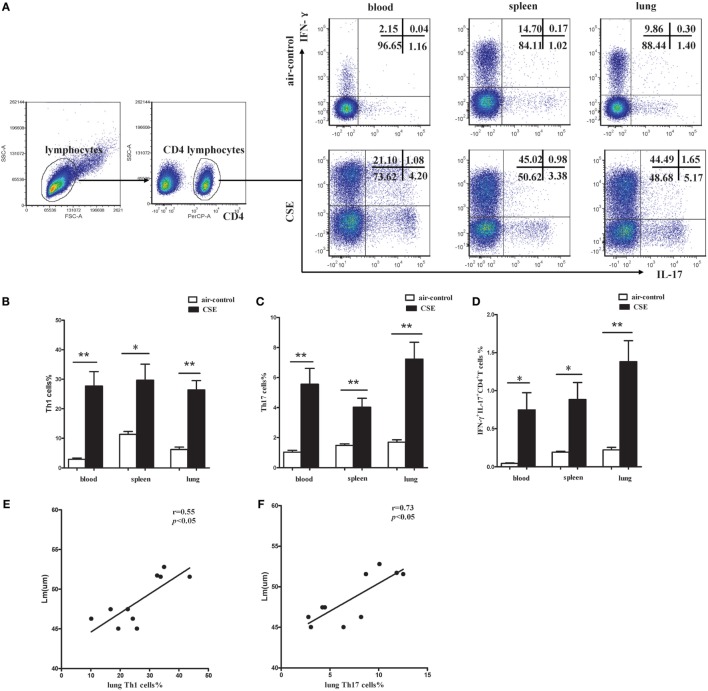
**Increased proportions of Th1 and Th17 cells in cigarette smoke-exposed mice**. **(A)** Lymphocytes were identified based on their characteristic properties shown in the FSC and SSC, and CD4^+^ T cells were gated from lymphocytes. The representative flow cytometric dot plots of Th1 and Th17 cells in peripheral blood, spleens, and lungs of air-control and cigarette smoke-exposed mice. **(B)** Comparisons of percentages of Th1 cells **(C)** and Th17 cells **(D)** IFN-γ^+^IL-17^+^CD4^+^ T cells in air-control and cigarette smoke-exposed mice. Data are expressed as means ± SD. The comparisons were determined by Student’s *t*-test (*p < 0.01, **p < 0.001). **(E)** Correlation between Lm and Th1 **(F)** and Th17 cells in lungs of cigarette smoke-exposed mice. Correlations between two parameters were determined by Spearman’s rank correlation test.

It is well known that activated CD4^+^ T cells participate in the pathogenesis of COPD *via* producing IFN-γ and other related cytokines. Regarding the CD4^+^ T cells from mice of cigarette smoke exposure exhibited an activated phenotype of CD62L^low^ and CD44^high^, we next focused on the cytokines secreted by activated CD4^+^ T cells. Flow cytometry was performed on CD4^+^ T cells after intracellular stained with anti-IFN-γ and anti-IL-17 mAbs (Figure 7A). In cigarette smoke-exposed mice, significant increases in both IFN-γ and IL-17 were observed in peripheral blood, spleens, and lungs compared with that in air-control mice (Figures 7B–D). Furthermore, the percentages of Th1 and Th17 cells in lungs of cigarette smoke-exposed mice were correlated positively with Lm (Figures 7E,F). These results collectively suggested that both Th1 and Th17 cells contribute to the pathogenesis of emphysema induced by cigarette smoke exposure.

### IL-27 Induced Th1 but Inhibited Th17 Differentiation *In Vitro*

**Figure 8:**
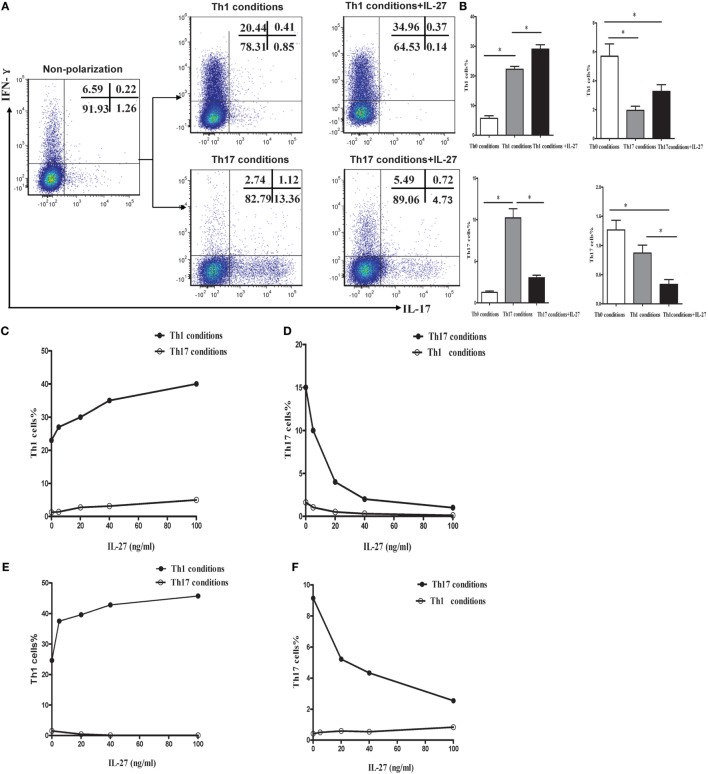
**IL-27 promoted the Th1 but inhibited the Th17 cells differentiation *in vitro***. **(A)** Naive CD4^+^ T cells from spleen of wild-type mice were cultured under Th1 and Th17 conditions in the presence or absence different concentrations of IL-27. The representative flow cytometric dot plots of Th1 and Th17 are showing from five independent experiments. **(B)** Comparisons of percentages of Th1 and Th17 cells in each group. The comparisons were determined by one-way ANOVA (*p < 0.05). **(C,D)** The effects of different concentrations of IL-27 on the cells under Th1 and Th17 conditions. **(E,F)** The effects of IL-27 on the cells after 6 days stimulated in Th1 and Th17 condition.

To explore the effects of IL-27 on the differentiation of Th1 and Th17 cells *in vitro*, naive CD4^+^ T cells were purified from mouse spleen and were cultured in Th1 or Th17 polarization conditions in the presence or absence of IL-27. Low to minimal IFN-γ and IL-17 was detected in naive CD4^+^ T cells before polarization. As presented in Figures 8A–C, *via* a dose-dependent manner, recombinant mouse IL-27 significantly enhanced the secretion of IFN-γ by CD4^+^ T cells in Th1 condition. Nevertheless, IL-27 can effectively suppress the production of IL-17 by CD4^+^ T cells in Th17 condition, this effect also in a concentration-dependent way (Figure 8D). In addition, we noted that the production of IFN-γ by CD4^+^ T cells in Th17 condition was also elevated after the administration of IL-27, but the increase was no statistically significant (Figure 8B).

Although only a small minority of effector CD4^+^ T cells expressing IL-27R (WSX-1), IL-27 might exert an immunological effect on these cells. To address this possibility, naive CD4^+^ T cells were isolated and stimulated in Th1 or Th17 polarization conditions. After cultured for 6 days, the activated cells were supplemented with recombinant mouse IL-27 and cultured for 2 more days. As shown in Figures 8E,F, IL-27 also promoted the production of IFN-γ by cells in Th1 condition but inhibited IL-17 by cells in Th17 condition *via* a dose-dependent way.

### Expression of T-bet and ROR-γt during Differentiation of Th1 and Th17

**Figure 9:**
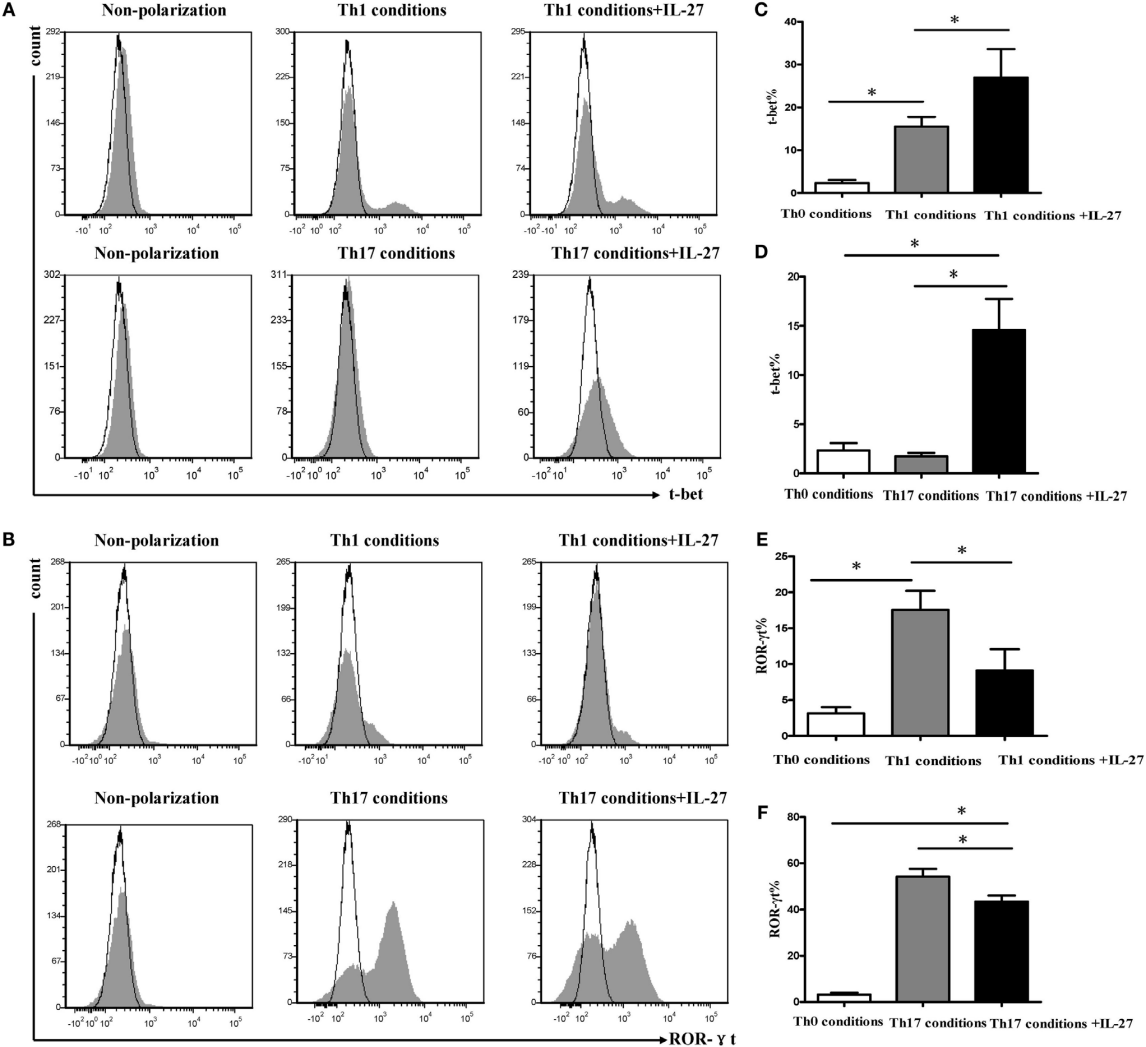
**Effects of IL-27 on expression of T-bet and ROR-γt during the differentiation of Th1 and Th17 cells**. Naive CD4^+^ T cells from spleen of wild-type mice were cultured under Th1 and Th17 conditions in the presence or absence different concentrations of IL-27. **(A,B)** The representative flow cytometric histogram-plot of T-bet or ROR-γt are showing from five independent experiments. **(C–F)** Comparisons percentages of T-bet and ROR-γt in each group. The comparisons were determined by one-way ANOVA (*p < 0.05).

Given T-bet and ROR-γt is the specific transcription factor of Th1 and Th17, which involves in their development and functions. We examined the expression of T-bet and ROR-γt during differentiation of Th1 and Th17 under the same polarization conditions. In agreement with the change in IFN-γ expression, supplement of IL-27 to cells under Th1 or Th17 condition significantly augmented the expression of T-bet (Figures 9A–D), whereas the addition of IL-27 to cells under Th17 condition markedly diminished the expression of ROR-γt (Figures 9B,E,F). Interestingly, we noted that about 10% of cells in Th1 conditions were expressed ROR-γt but only a minor fraction of T-bet was expressed on the cells in Th17 conditions (Figures 9D,E).

### STAT Signals Involved in the Differentiation of Th1 and Th17

**Figure 10:**
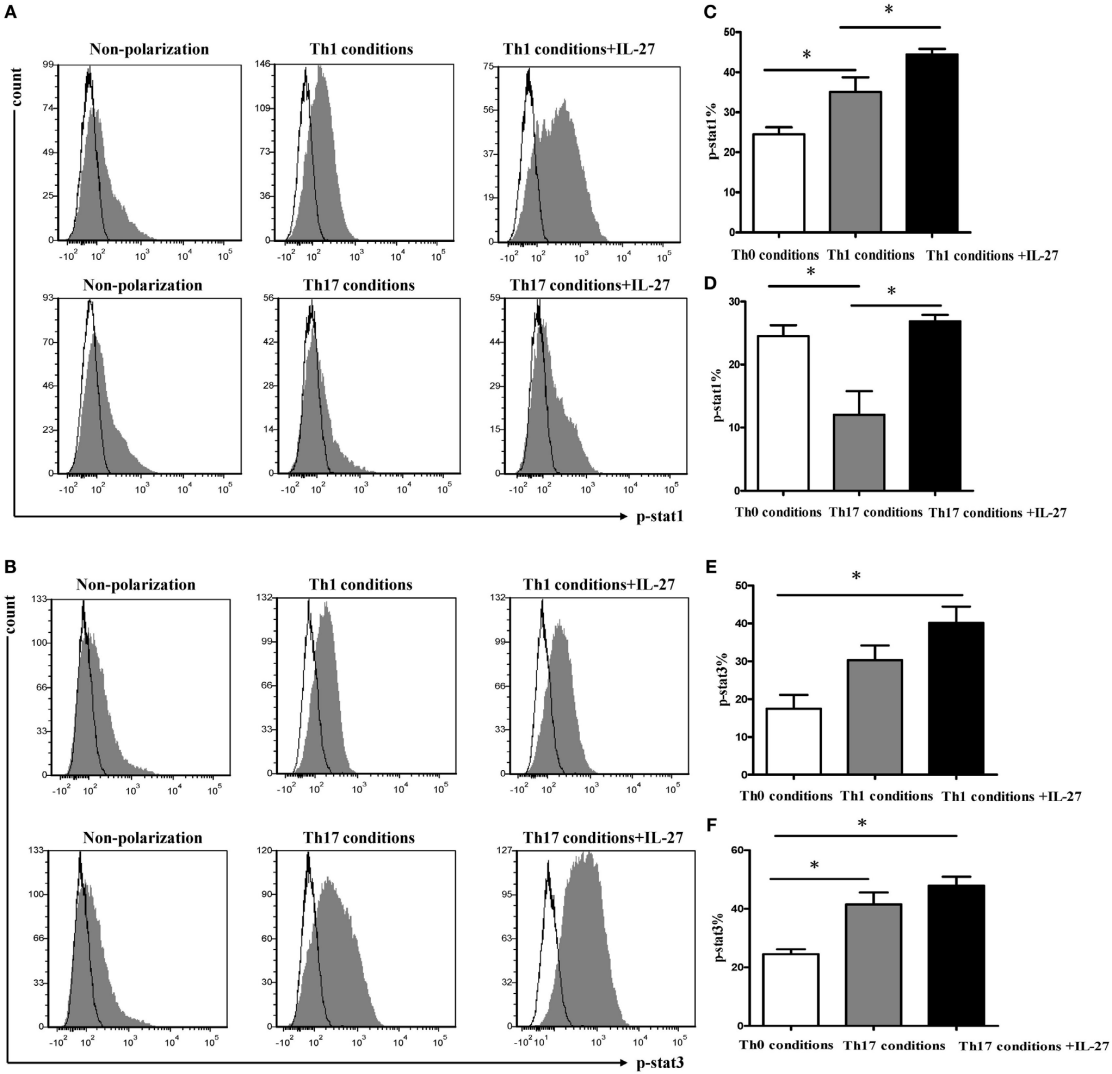
**Signal transductions involved in differentiation Th1 and Th17 cells**. Naive CD4^+^ T cells from spleens of wild-type mice were cultured under Th1 and Th17 conditions. Three days later, the medium was supplemented with different concentrations of IL-27 and the cells were stimulated for 30 min and intracellular stained with p-STAT1, p-STAT3. **(A,B)** The representative expression of p-STAT1, p-STAT3 in cells under Th1 and Th17 conditions in the presence or absence of IL-27. **(C–F)** Comparisons of percentages of p-STAT1, p-STAT3 in each group. The comparisons were determined by one-way ANOVA (*p < 0.05).

We noted that STAT1 and STAT3 were both involved in the differentiation of Th1 cells, and the addition of IL-27 triggered a rapid and significant increase in STAT1 and STAT3 phosphorylation (Figures 10A–C,E). Similar results were observed in the cells under Th17 conditions. Although the cells in Th17 condition with a higher extent of STAT3 activation (Figures 10B,F), supplement of IL-27 to mediums caused a significant increase in STAT1 phosphorylation (Figures 10A,D).

### Effects of IL-27 Neutralization on Cigarette Smoke-Exposed Mice

**Figure 11:**
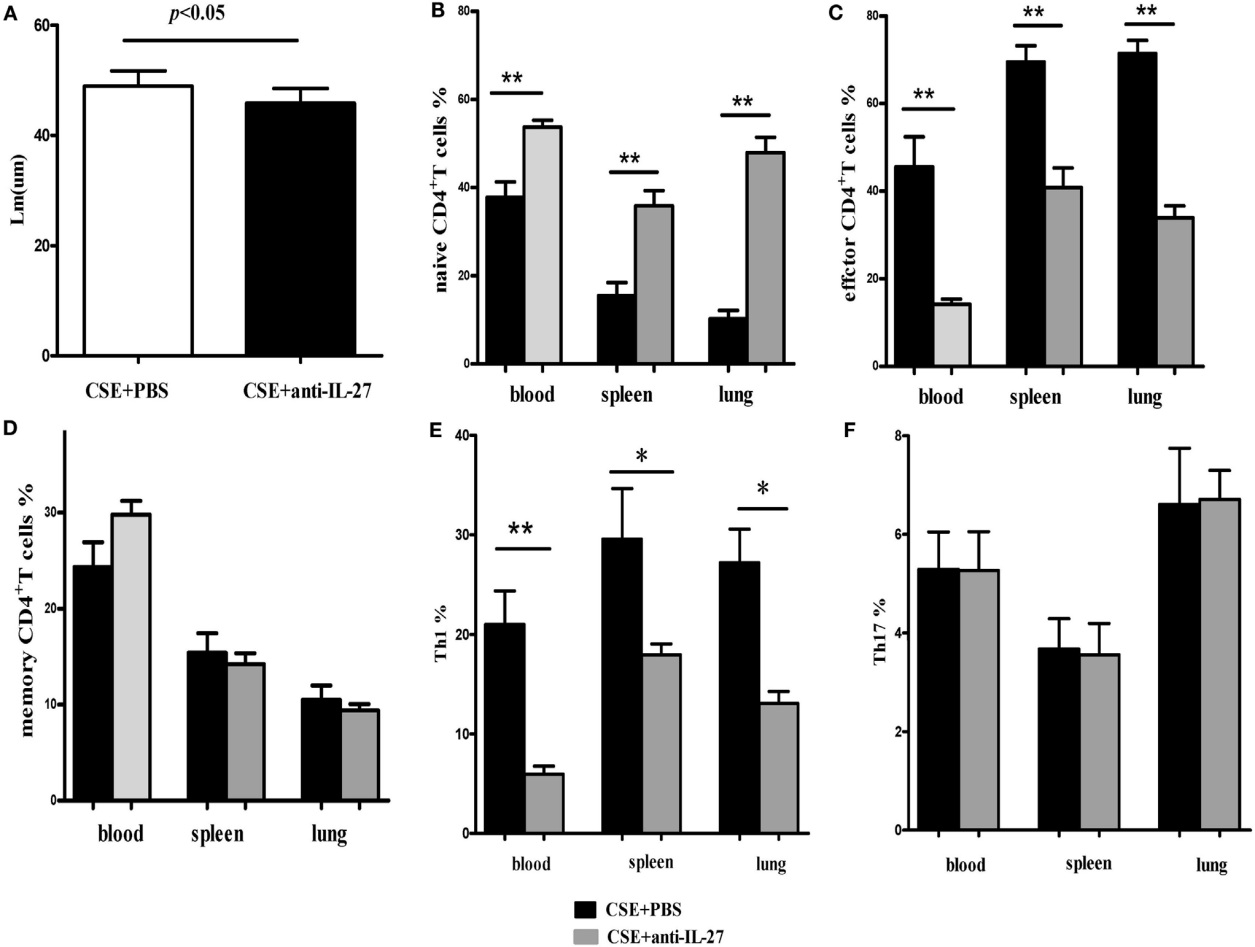
**Anti-IL-27 treatment decreased the proportions of effector CD4^+^ T cells and downregulated the expression of Th1 cells in cigarette smoke-exposed mice**. Mouse was treated by tail vein injection with anti-mouse IL-27 p28 functional grade purified mAbs twice a week (10 μg in 200 μl PBS per time) in the course of cigarette smoke exposure. **(A)** Comparisons of Lm values in cigarette smoke-exposed mice treated with anti-IL-27 antibody and in mice treated with PBS. **(B)** Comparisons of percentages of naive CD4^+^ T cells **(C)** and effector CD4^+^ T cells **(D)** and memory CD4^+^ T cells **(E)** and Th1 cells **(F)** and Th17 cells in cigarette smoke-exposed mice treated with anti-IL-27 antibody and mice treated with PBS (each *n* = 8). Data are expressed as means ± SD. The comparisons were determined by Student’s *t*-test (*p < 0.05, **p < 0.001).

Finally, we determined the effects of anti-IL27 treatment on the development of Th1 and Th17 cells *in vivo*. We injected anti-mouse IL-27 p28 functional grade purified mAbs into mice intravenously twice a week in the course of cigarette smoke exposure. We observed that the emphysema in mice receiving anti-IL-27 treatment was ameliorated to a certain extend as compared with which received PBS (Figure 11A). The total number of cells isolated from spleen and right lung were showed in Table 2. We confirmed the neutralization effect by measuring the concentrations of serum IL-27 in mice which received anti-IL-27 treatment with ELISA (Figure S1C in Supplementary Material). As expected, anti-IL-27 treatment induced a higher proportion of naïve CD4^+^ T cells but a lower frequency of effector CD4^+^ T cells in cigarette smoke-exposed mice when compared with mice treating with PBS (Figures 11B,C). The proportion of memory CD4^+^ T cells was no statistical difference between two groups (Figure 11D). In addition, treatment with anti-IL-27 antibody attenuated the expression of Th1 cells in cigarette smoke-exposed mice (Figure 11E). However, the expression of Th17 cells in mice that treated with anti-IL-27 antibody was not significantly different from that of control mice (Figure 11F).

**Table 2:** **Total number cells of spleen and lung**.

	CSE + PBS (*n* = 8)	CSE + anti-IL-27 (*n* = 8)
Spleen cells (×10^7^)	11.76 ± 1.63	12.40 ± 1.51
Lung cells (×10^7^)	2.86 ± 0.98	2.76 ± 0.86

*Data are expressed as means ± SD*.

## Discussion

Chronic inflammation in the airways and the lung parenchyma which mainly triggered by cigarette smoke is the most remarkable characteristic of COPD. Th1 and Th17 cells have been considered to be key driver and effector cells in this chronic inflammatory procedure, but the immune regulation between these cells remains elusive. Here, we demonstrated that elevated IL-27 was associated with increased expression of Th1 and Th17 cells in patients with COPD. In addition, cigarette smoke exposure dramatically upregulated the expression of IL-27R (WSX-1) by naive CD4^+^ T cells in a mouse model of emphysema. Furthermore, IL-27 significantly augmented the secretion of IFN-γ but inhibited the production of IL-17 by naive CD4^+^ T cells *in vitro*. More importantly, anti-IL-27 treatment attenuated the expression of IFN-γ-producing CD4^+^ T cells in cigarette smoke-exposed mice. These results suggested that IL-27 regulates the development and differentiation of Th1 and Th17 cells and may be a reason responsible for the exaggerated Th1 response in COPD.

A large body of evidence has established that Th1 cells play a pivotal role in the pathogenesis of COPD. Recent literatures proposed that Th17 cells may involve in the development and progression of the disease. In the present study, we observed Th1 and Th17 cells both were markedly increased in peripheral blood of COPD as compared with the healthy smokers and non-smokers. Consistent with Cao et al. reports (23), we showed that the protein level of IL-27 in serum was significantly elevated in COPD. In addition, the serum concentrations of IL-27 in COPD were correlated positively with the percentages of Th1 cells, suggesting a possible connection between IL-27 and these cells. Additionally, early studies have reported there are interesting parallels between Th1 and Th17 lineage developmental programs. In this regard, it is possible to predict IL-27 might exert potential effect on Th17 cells differentiation. But the underlying mechanisms by which IL-27 regulation of Th1 and Th17 cells in smoking-related COPD still unknown.

By employing a mouse model of emphysema induced by cigarette smoking, we achieved results similar to COPD patients that not only IL-27 protein but also p28 and EBI3 mRNA expression was notably upregulated in mice after cigarette smoke exposure. IL-27 executes its function through binding to its receptor complex (IL-27R). There were reports documented that WSX-1 mRNAs was markedly upregulated within the brain lesions of postmortem multiple sclerosis or in inflammatory cells in the CNS during EAE (19, 25). Additionally, Villarino and colleagues found that the expression of WSX-1 was increased during acute toxoplasmosis (26). But to the best of our knowledge, there were no data available so far concerning the expression of IL-27R in cigarette smoke-exposed mice. In the current study, our results revealed that WSX-1 was mainly expressed on naive but not effector or memory CD4^+^ T cells in line with previous reports (data no shown), implying that naïve CD4^+^ T cells were the major target of IL-27 action within the smoking induced inflammation. Importantly, we demonstrated for the first time that IL-27R (WSX-1) was upregulated on naïve CD4^+^ T cells in the setting of cigarette smoke-induced emphysema, which absence on effector T cells. In view of this, it was plausible to conclude that the upregulation of IL-27 and IL-27R on naïve CD4^+^ T cells could render naive CD4^+^ T cells differentiate into a specific T cell linage in the stimulation of cigarette smoke. As expect, we observed a majority of CD4^+^ T cells in mice of cigarette smoke exposure displayed the phenotype of effector and memory cells. Moreover, a considerable increase of IFN-γ and IL-17 in CD4^+^ T cells was found after cigarette smoke exposure. Therefore, these findings gave rise to speculation that IL-27 triggered by cigarette smoke exposure might interact with IL-27R on naive CD4^+^ T cells, thus contribute to naive CD4^+^ T cells differentiate into IFN-γ-producing effector CD4^+^ T cells in smoking mouse model of emphysema.

IL-27 has been found to possess pro- and anti-inflammatory activity and play an essential role during the pathogenesis of infectious and autoimmune diseases (27–30), but its functions in chronic inflammatory disease remain controversial. The initial reports described IL-27 as a promoter of Th1 polarization and IFN-γ production of naive CD4^+^ T cells (12). In contrast, recent studies found that IL-27 receptor signaling exerted its anti-inflammatory activities by preventing Th1-mediated immunopathology during *T. gondii* and malaria infection (18, 31–33). In addition, there were reports showed that IL-27 negatively regulated the development of Th17 during chronic inflammation of the central nervous system in mice that chronically infected with *T. gondii*, or in mice that coxsackievirus-B3-induced viral myocarditis (20, 21, 34). In light of substantially increased IL-27 in the mice after cigarette smoke exposure, we investigated the role of IL-27 in the differentiation of Th1 and Th17 cells *in vitro*. The result demonstrated that *via* a dose-dependent manner, recombinant mouse IL-27 significantly promoted the secretion of IFN-γ but inhibited the production of IL-17 by naive CD4^+^ T cells in Th1 conditions or in Th17 conditions. In addition, we noted that even in cells that had been committed in Th1 or Th17 lineage in which TCCR expression was substantially diminished; IL-27 still remained functions for enhancing the Th1 differentiation and depressing Th17 differentiation. Since previous studies focused on the effects of IL-27 on naive CD4^+^ T cells, this observation may expand the role of IL-27 in other subgroup of CD4^+^ T cells. These data collectively identified a dominant function for IL-27 in the induction of Th1 response from naive CD4^+^ T cells *in vitro*, and the potential effect of IL-27 on antagonizing Th17 response.

T-bet is the specific transcription factor, which programs the differentiation of Th1 cells and ROR-γt, is the key transcription factor that orchestrates the differentiation of Th17 cells. Earlier researches have discovered that IL-27 induced the expression of T-bet during initial Th1 commitment but blocked ROR-γt expression to inhibit the lineage commitment of Th17 cells (35, 36). In agreement with previous studies, we found IL-27 significantly augmented T-bet expression by naive CD4^+^ T cells in Th1 conditions or in Th17 conditions. In contrast, the expression of ROR-γt was dramatically suppressed by the addition of IL-27 in Th1 conditions or in Th17 conditions. Th17 cells appeared to have greater plasticity in their late stages of development, which stimulated with IL-12 induced a rapid transition of IFN-γ expression *via* a STAT4- and T-bet-dependent manner, whereas Th1 cells are more resistant to transition to a Th17 cytokine-expression pattern (37–39). In this study, as the presence of IL-17^+^IFN-γ^+^CD4^+^ T cells, we observed the coexpression of T-bet and ROR-γt in cells whatever under Th1 condition or Th17 condition, suggesting there are parallels between the developmental programs of Th1 and Th17 cells. Stimulation of cells under Th17 conditions with IL-27, leading to an increase expression of T-bet accompanied by the excessive secretion of IFN-γ. However, the cells already polarized to Th17 without shifted their phenotype toward enhanced IFN-γ production in the presence of IL-27 alone. Further studies are needed to address the impact of the combination of IL-27 with IL-12 on the transition of Th17 to IFN-γ secretion pattern; given that IL-27 and IL-12 are abundant in COPD.

Although the activation of STAT1 was linked with IL-27-induced Th1 polarization (35, 40), our data provided evidence that STAT3 was also participated in this procedure. In the current study, both p-STAT1 and p-STAT3 were increased by the addition of IL-27 into cells under Th1 condition, which in line with the expression of T-bet and IFN-γ, suggesting the activation of STAT1 and STAT3 are all mediating T-bet induction in the course of IL-27 initiated Th1 commitment. This result is in agreement with a study by Jung et al., which found that elevated IL-27 levels in primary human neonatal macrophages regulated indoleamine dioxygenase in a STAT-1 and STAT-3-dependent manner (41). Activation of STAT3 by IL-6 and IL-23 drives the production of IL-17 by CD4^+^ T cells. Nevertheless, the function of IL-27 on inhibition of IL-17 production by CD4^+^ T cells was substantial compromised in absence of STAT1 (21). As shown in this study, we found that p-STAT3 was predominating in Th17 conditions, whereas the addition of IL-27 elicited a pronounced increase of p-STAT1, but not p-STAT3. This observation supported the idea that phosphorylation of STAT1-mediates inhibition activity of IL-27 to Th17 cells.

It has been reported that IL-27 plays an anti-inflammatory role in septic mice and contributes to the sepsis-induced immuno-suppression, blockading the biological function of IL-27 results in increased survival and reduces the susceptibility to secondary *P. aeruginosa* infection (42, 43). However, in contrast to the findings in sepsis, the increased expression of IL-27 in COPD was accompanied with an amplified, uncontrolled chronic inflammatory response, suggesting a pro-inflammatory activity of IL-27 in this context. Thus, we assessed the potential effects of neutralizing the biological function of IL-27 in cigarette smoke-exposed mice. We observed that anti-IL-27 treatment effectively decreased Th1-mediated inflammation in cigarette smoke-exposed mice. Interestingly, there was no significant difference in Th17 cells expression between mice treating with anti-IL-27 antibody and mice treating with PBS. Despite the inhibitory activity of IL-27 on the secretion of IL-17 by naive CD4^+^ T cells *in vitro*, IL-27 neutralization did not lead to a more exaggerated IL-17 production in smoking mouse model of emphysema. It seems that there are other mediators antagonize Th17 response or the residual IL-27, which have not been extinguished by anti-IL-27 treatment still exerts fully function on Th17 cells. Therefore, further studies (e.g., using IL-27R alpha/WSX-1^−/−^ mice model) are needed to determine the role of IL-27 on Th17 cells *in vivo*.

In conclusion, here, we have demonstrated that IL-27 was elevated in patients with COPD and in a mouse model of emphysema. In addition, cigarette smoke exposure induced the upregulation of IL-27R (WSX-1) by naive CD4^+^ T cells in cigarette smoke-exposed mice. IL-27 has the function for promoting the expression of Th1 cells but inhibiting the expression of Th17 cells *in vitro*. *In vivo*, IL27 neutralization significantly attenuated Th1-mediated inflammation. Thus, our findings contribute to a better understanding of pathogenesis of emphysema induced by cigarette smoke exposure, and targeting IL-27/WSX-1 may provide a new therapeutic approach for COPD.

## Author Contributions

M-CD designed the study design and the experimental protocols. S-LQ, YL, and H-JT were responsible for flow cytometry and data collection. S-LQ, L-MZ, and C-MY were responsible for cell isolation and culture. G-NL and M-CD analyzed the data. S-LQ and M-CD drafted the manuscript. All the authors read, critically revised, and agreed to be accountable for the content of the work.

## Conflict of Interest Statement

The authors declare that the research was conducted in the absence of any commercial or financial relationships that could be construed as a potential conflict of interest.
