# Supplementary material for: Cigarette Smoke Induction of Interleukin-27/WSX-1 Regulates the Differentiation of Th1 and Th17 Cells in a Smoking Mouse Model of Emphysema
Source: Front Immunol. 2016 Dec 5;7:553. doi: 10.3389/fimmu.2016.00553 (PMC5136545; doi:10.3389/fimmu.2016.00553)
Supplement: Supplementary file 1 [file Image_1.PDF]

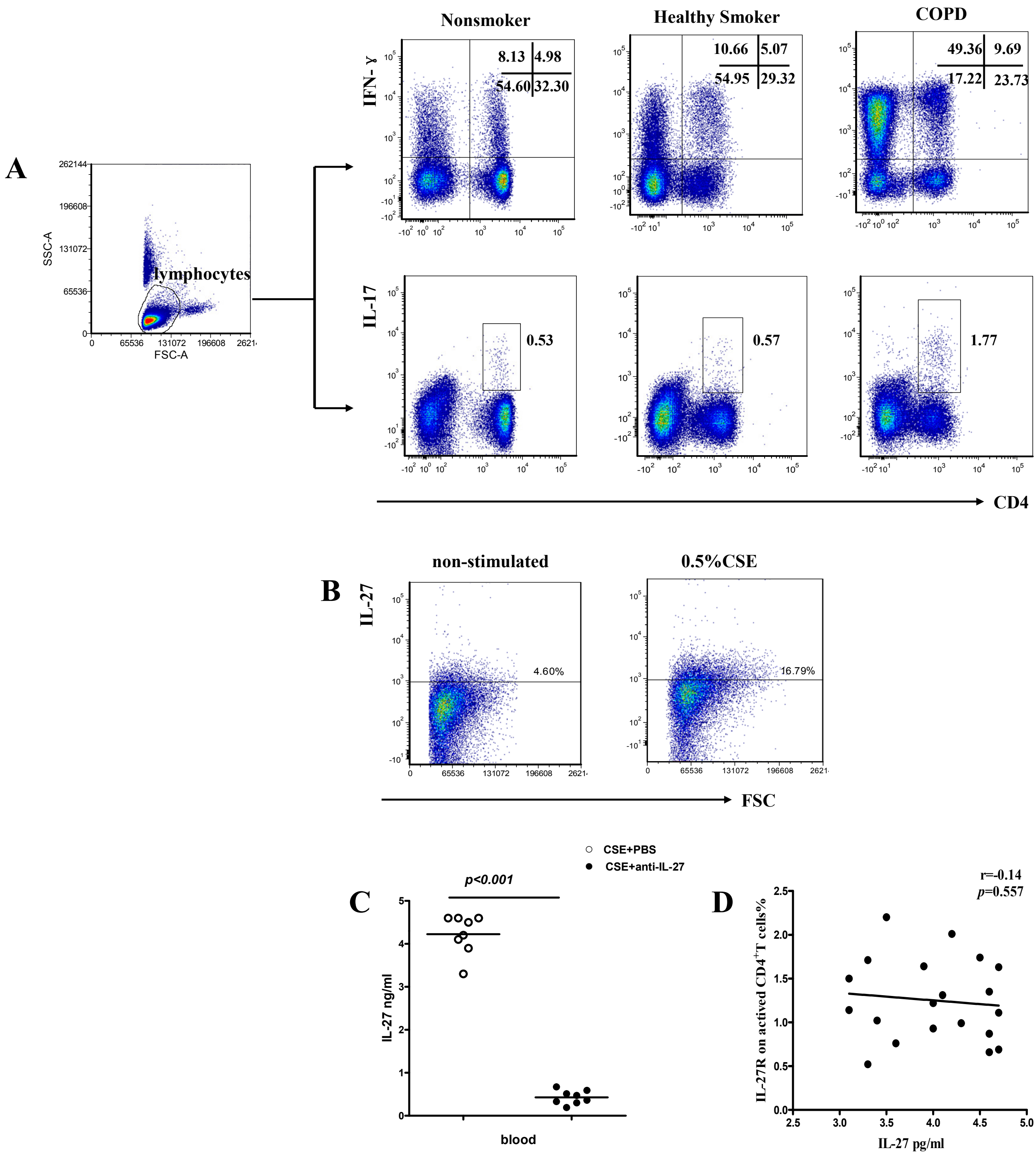

Figure S. (A) The proportion of IFN-γ-producing CD4+T(Th1) cells and IL-17-producing CD4+T (Th17) cells in total lymphocytes in peripheral blood of COPD patients. (B) CSE promoted the production of IL-27 by bone marrow-derived dendritic cells (mDCs) derived from mouse. (C) The concentrations of serum IL-27 in mice receiving PBS and anti-IL-27 treatment. (D) The correlation between activated CD4 T cells IL-27R expression and IL-27 concentration in spleens and lungs of cigarette smoke exposed mice.
